# COVID-19 and assisted reproductive technology services: repercussions for patients and proposal for individualized clinical management

**DOI:** 10.1186/s12958-020-00605-z

**Published:** 2020-05-13

**Authors:** Carlo Alviggi, Sandro C. Esteves, Raoul Orvieto, Alessandro Conforti, Antonio La Marca, Robert Fischer, Claus Y. Andersen, Klaus Bühler, Sesh K. Sunkara, Nikolaos P. Polyzos, Ida Strina, Luigi Carbone, Fabiola C. Bento, Daniela Galliano, Hakan Yarali, Lan N. Vuong, Michael Grynberg, Panagiotis Drakopoulos, Pedro Xavier, Joaquin Llacer, Fernando Neuspiller, Marcos Horton, Matheus Roque, Evangelos Papanikolaou, Manish Banker, Michael H. Dahan, Shu Foong, Herman Tournaye, Christophe Blockeel, Alberto Vaiarelli, Peter Humaidan, Filippo M. Ubaldi, on behalf of the POSEIDON (Patient-Oriented Strategies Encompassing IndividualizeD Oocyte Number) group

**Affiliations:** 1 Department of Neuroscience, Reproductive Science and Odontostomatology, University of Naples Federico II, Via Sergio Pansini 5, 80131 Naples, Italy; 2 ANDROFERT, Andrology and Human Reproduction Clinic, Av. Dr. Heitor Penteado, 1463, Campinas, SP 13075-460 Brazil; 3 Department of Surgery (Division of Urology), University of Campinas (UNICAMP), Campinas, Brazil; 4 Faculty of Health, Aarhus University, Aarhus, Denmark; 5 Department of Obstetrics and Gynecology, Chaim Sheba Medical Center, Ramat Gan, Israel; 6 The Tarnesby-Tarnowski Chair for Family Planning and Fertility Regulation, Sackler Faculty of Medicine, Tel-Aviv University, Tel Aviv, Israel; 7 Department of Medical and Surgical Sciences for Children and Adults, University of Modena and Reggio Emilia, Modena, Italy; 8 Fertility Center Hamburg, Hamburg, Germany; 9 Laboratory of Reproductive Biology, University Hospital of Copenhagen, Faculty of Health and Medical Sciences, Copenhagen, Denmark; 10 Center for Gynecology, Endocrinology and Reproductive Medicine, and Stuttgart, Ulm, Germany; 11 Scientific-Clinical Centre for Endometriosis of the University Hospitals of Saarland, Saarbrücken, Germany; 12 Department of Women’s Health, Faculty of Life Sciences, King’s College London, London, UK; 13 Dexeus University Hospital, Barcelona, Spain; 14 Instituto Valenciano de Infertilidad (IVI), Rome, Italy; 15 Anatolia IVF, Ankara, Turkey; 16 Department of Obstetrics and Gynecology, University of Medicine and Pharmacy at Ho Chi Minh City, Ho Chi Minh City, Vietnam; 17 IVFMD, My Duc Hospital, Ho Chi Minh City, Vietnam; 18 Service de Médecine de la Reproduction et Préservation de la Fertilité, Hôpital Antoine Béclère, Clamart, France, Université Paris Saclay, Saint-Aubin, France; 19 Center for Reproductive Medicine, Universitair Ziekenhuis Brussel, Vrije Universiteit Brussel, Brussels, Belgium; 20 Department of Obstetrics and Gynecology, Medical School, University of Crete, 71110, Heraklion, Crete, Greece; 21 Unit of Reproductive Medicine, Department of Gynecology and Obstetrics, Hospital Center São João, Porto, Portugal; 22 Instituto Bernabeu, Alicante, Spain; 23 Instituto Valenciano de Infertilidad (IVI), Buenos Aires, Argentina; 24 Pregna Medicina Reprodutiva, Buenos Aires, Argentina; 25 MaterPrime, São Paulo, Brazil; 26 Assisting Nature, Centre of Assisted Reproduction and Genetics, Thessaloniki, Greece; 27 3rd Department of Obstetrics and Gynecology, Aristotle University of Thessaloniki, Thessaloniki, Greece; 28 Nova IVF Fertility, Ahmedabad, India; 29 Department of Obstetrics and Gynecology, McGill University, Montreal, Canada; 30 Department of Obstetrics & Gynecology, University of Calgary, Calgary, Canada; 31 Regional Fertility Program, Calgary, Canada; 32 Fertility Clinic Skive, Skive Regional Hospital, Skive, Denmark; 33 GENERA, Center for Reproductive Medicine, Rome, Italy

**Keywords:** COVID-19, Assisted reproductive technology, Infertility, In vitro fertilization, Intracytoplasmic sperm injection, Poseidon criteria, Viewpoint

## Abstract

The prolonged lockdown of health services providing high-complexity fertility treatments –as currently recommended by many reproductive medicine entities– is detrimental for society as a whole, and infertility patients in particular. Globally, approximately 0.3% of all infants born every year are conceived using assisted reproductive technology (ART) treatments. By contrast, the total number of COVID-19 deaths reported so far represents approximately 1.0% of the total deaths expected to occur worldwide over the first three months of the current year. It seems, therefore, that the number of infants expected to be conceived and born –but who will not be so due to the lockdown of infertility services– might be as significant as the total number of deaths attributed to the COVID-19 pandemic. We herein propose remedies that include a prognostic-stratification of more vulnerable infertility cases in order to plan a progressive restart of worldwide fertility treatments. At a time when preventing complications and limiting burdens for national health systems represent relevant issues, our viewpoint might help competent authorities and health care providers to identify patients who should be prioritized for the continuation of fertility care in a safe environment.

## Background

Recently, governments around the world announced the most far-reaching restrictions of personal freedom in modern history due to COVID-19. The remarkable increase in COVID-19 cases raises the prospect of massive hospitalizations that no healthcare system in the world can manage. The urgent need to avoid this scenario is the justification for the implemented restrictions, and reproductive medicine societies decisively followed by issuing expert guidance based best judgment. With a solid consensus, the key recommendations for practitioners include suspension of new fertility treatments –ovulation induction, intrauterine insemination (IUI), and in vitro fertilization (IVF)– as well as non-urgent gamete cryopreservation, cancellation of all embryo transfers, whether fresh or frozen and suspension of elective surgery and non-urgent diagnostic procedures [1, 2]. Exceptions are those patients who are currently ‘in-cycle’ or who require urgent fertility preservation due to cancer treatment.

We agree that faced with increasing numbers of coronavirus infections across the world, no medical society would have acted differently. However, taking the above mentioned into account, we would like to raise a novel and constructive viewpoint. Our concern is that a prolonged lockdown of fertility treatment will be detrimental to both patients and society. Moreover, the fertility community is uncertain about how to optimally provide care to infertile patients –without compromising safety– once the restart of fertility services is established. Thus, we aim at proposing remedies to mitigate the long-term consequences of a prolonged cessation of infertility treatment and to help regulatory authorities and health care providers identify which patients might be prioritized for the continuation of fertility care in a safe environment.

### The pandemic facts

At the time of writing (April 23), the global COVID-19 deaths represent approximately 1.0% of total deaths expected to occur worldwide over the first 3 months of the current year, with a wide variation in the reported death rates per country, ranging from 3% in Germany to 13% in Italy [3]. In total, more than two million COVID-19 cases have been reported, 97% of which have been defined as mild. Among the severe or critical cases, the overwhelming majority affects people aged 50 and above. By contrast, the death rate among people at reproductive age is 0.2%, with an estimated 1.5:1 male to female ratio, mainly affecting those with pre-existing conditions, including cardiovascular disease, diabetes, chronic respiratory disease, obesity, hypertension, and cancer [3] .

### The interpretation and impact for patients undergoing assisted reproductive technology treatment

While it is prudent to advocate temporary social distancing and closure of non-emergency health services, we would like to point at what a prolonged lockdown of fertility treatment might mean for the society as a whole, and infertility patients in general. This consideration will focus on assisted reproductive technology (ART) treatment, particularly in the low prognosis patients who represent approximately 30–50% of patients seeking ART [4].

Conservative estimates indicate that over 1.5 million IVF cycles are carried out every year worldwide, resulting in approximately 400,000 babies born [5]. In the United Kingdom and the United States, approximately 3 and 2%, respectively, of all infants born every year are conceived using ART [6, 7]. Globally, ART babies represent about 0.3% of the total live birth rate every year [5, 8]. Presently, we do not know how long the suspension of fertility treatments will last; however, estimates ranging from 3 to 12 months or even longer have been suggested, depending on how effective governments implement quarantine measures and how long it takes to acquire herd immunity. Thus, the number of infants expected to be conceived and born –but who will be so due to the lockdown of infertility services– might be as significant as the total number of deaths attributed to the COVID-19 pandemic.

Along these lines, the ‘time’ variable is crucial for specific subgroups of infertile women, in particular, patients with ‘low prognosis’ for success in ART who tends to lose their fertility potential rapidly. The implications of postponing ART treatment under these circumstances demand significant attention. Notably, the probability of embryo euploidy, i.e., having an exact multiple of the haploid number of chromosomes, declines sharply after the age of 34 years and is overall lower than 50% for this subgroup (Supplementary Figure 1) [9]. At ages 35, 36, 37, 38, 39, and 40 years, the relative loss in embryo euploid probability from the previous year is 6.7, 8.2, 9.8,11.6, 13.6, and 15.7%, respectively (Supplementary Figure 2) [9].

The impact of female age on the success rates of ART is more dramatic in older than younger patients as more oocytes are needed –in the former– to obtain at least one euploid embryo for transfer.

Along these lines, the ART calculator –a clinical predictive model used to estimate the number of mature (metaphase II) oocytes needed to obtain at least one euploid embryo in couples undergoing ART – indicates that 13 (95% confidence interval [CI]: 11–16), 16 (95% CI: 13–20), and 19 (95% CI: 15–25) oocytes are needed for women aged 38, 39, and 40 years, respectively [10, 11]. By contrast, only 5 (95% CI: 4–6) and 6 (95% CI: 5–7) oocytes are required for patients aged 33 and 34 years to have at least one euploid embryo [10, 11]. These figures, combined with the proven reduced ovarian reserve in patients of advanced age, clearly indicate that reproductive outcomes will be decreased if treatment is further delayed.

The Poseidon group in 2015 introduced new criteria to identify the ‘low prognosis patient’ undergoing ART [12–14]. Based on female age, ovarian reserve, and previous history of ovarian stimulation –if available–, ‘low prognosis’ patients were stratified into four specific subgroups, each of which with a distinct prognosis concerning the likelihood of conceiving and delivering a baby with the use of ART (Supplementary Figure 3). The primary aim of the Poseidon criteria was to provide patient counselling and clinical guidance regarding interventions that could help these patients conceive in the shortest possible time. The most vulnerable Poseidon patients are those within groups 2 and 4, i.e., ageing patients 35 years or above, with (i) an unexpected suboptimal number of oocytes retrieved in a previous ART cycle despite a normal ovarian reserve (group 2), or (ii) low ovarian reserve in whom the number of oocytes resulting from ovarian stimulation is invariably low (group 4) [12, 15, 16]. The reason relates to the impact of maternal age and number of oocytes on the availability of euploid embryos, and thus, live birth. In particular, Poseidon group 4 patients are running out of time, and any further delay in treatment might imply the loss of a future opportunity to obtain a biological child. Indeed, the percentage of initiated ART cycles resulting in a live birth is estimated to be 41.5% in women younger than 35 years; however, these numbers dramatically decrease with ageing, being less than 5% in women above 40 years [17].

### The impact of COVID-19 for women in need of fertility preservation

Women affected by cancer or other conditions (e.g.., autoimmune disorders, systemic lupus erythematosus, hematologic disorders), who need to undergo gonadotoxic treatment, require urgent procedures for fertility preservation [18, 19]. Immediate oocyte or embryo cryopreservation could represent the only opportunity to obtain a pregnancy after the end of gonadotoxic treatment. At present, the prevailing consensus is to allow fertility preservation programs to continue for oncological patients. On the other hand, more prudence concerning embryo transfer is proposed. Notably, as new scenarios emerge, even embryo transfer could be challenging to postpone in some specific subgroups of patients. For instance, in women with endocrine responsive breast cancers, an international panel of oncologists have proposed a temporary interruption in endocrine therapy to allow patients to obtain natural or assisted conception [20, 21]. In these women, the full course of endocrine therapy, which for some cases might be at least 5–10 years, could dramatically decrease natural fecundity after finalisation of therapy. According to the POSITIVE trial approach, the therapeutic suspension ‘window’ should not be longer than 24 months to allow patients to conceive and deliver [20]. If no conception occurs, patients must restart adjuvant therapy. If such an approach is validated, and importantly, the trial is still ongoing, every strategy to obtain a pregnancy within this short timeframe, including IVF and embryo transfer, should be considered without further delay.

Besides oncologic disorders, other conditions might require fertility preservation. A classic example includes systemic autoimmune diseases (SADs), which are not uncommon in women of reproductive age. In these patients, the chronic inflammation might affect the hypothalamic-pituitary-ovarian axis and the ovaries directly, causing impairment of the ovarian reserve [22–24]. Moreover, gonadotoxic drugs (e.g., alkylating agents) and teratogenic agents (e.g., Mycophenolate mofetil) are commonly used to control the inflammatory processes in patients with SADs. In these patients, fertility preservation is conditioned to the “remission window” [25]. In other words, the inflammatory process has to be restrained before ovarian stimulation and pregnancy can be considered. Thus, it has been recommended that women with SADs (e.g., systemic lupus erythematosus, antiphospholipid antibody syndrome, rheumatoid arthritis, systemic sclerosis, Sjögren’s syndrome, mixed connective tissue disease, idiopathic inflammatory myopathies and vasculitis, among others) only embark on ART after at least 6 months of clinical remission has elapsed [25]. Several patients with SADs seeking fertility might have been planning this ‘window’ for a considerable time, which unfortunately occurred during the COVID-19 crisis –postponing IVF in these patients compromises their reproductive chances considerably.

Lastly, young patients with a diminished ovarian reserve and being at risk of premature ovarian insufficiency (i.e., Poseidon group 3) also represent a “time-sensitive” category of women [15, 26]. Due to the fact that Poseidon group 3 patients are younger than 35 years and, therefore, the oocyte/embryo euploidy rate is over 50%, the number of oocytes required to have at least one euploid embryo in these patients is lower than in older patients (e.g., 35 years and older) in whom the oocyte/embryo euploidy rate is below 50% overall (e.g., Poseidon groups 2 and 4). Nevertheless, it is not unlikely that when the ovarian reserve is dramatically reduced, the remaining window to respond to ovarian stimulation is narrow. In reality, this scenario is not very different from the pre-chemotherapy situation. On this basis, we believe that Poseidon group 3 patients should be allowed the same permissive approach for fertility preservation as suggested for women who need to undergo chemotherapy.

### Possible remedies for restarting ART treatments

First of all, the health and psychological consequences of not only offering the above subgroups of patients ART but also the impact on the resulting pregnancy need to be taken into consideration. Besides, male patients undergoing medical treatment aiming at improving semen quantity or quality should not be forgotten. Like men at reproductive age with cancer who are recommended to freeze sperm urgently, these patients should also be allowed to cryopreserve sperm or to even fertilize oocytes for subsequent embryo cryopreservation. Thus, andrological services must be are available for them too. Secondly, it might take months, or even years, before we can assess the broader implications of the current restrictive measures for patients as well as society. The damage to the affected patients is difficult to ascertain; however, removing infertility services for those who need it most might be even worse than the risks of treating them at the current time. While the various lockdowns have slowed the spread of infection, new cases will appear as soon as measures are loosened.

**Fig. 1:**
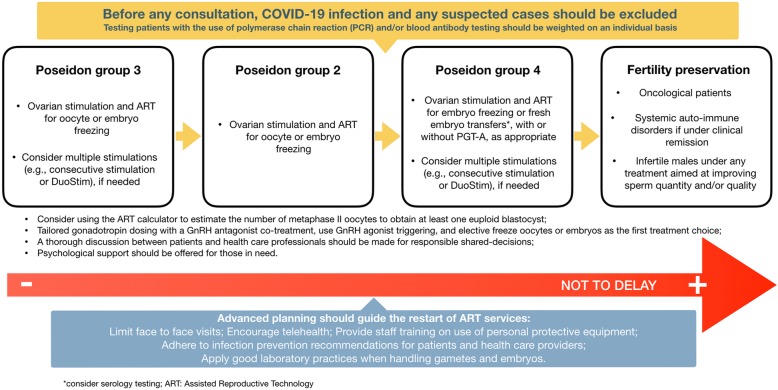
COVID − 19 and Assisted Reproductive Technology: proposal for individualized clinical management

In this time of uncertainty, we propose some remedies that we believe give fertility providers and patients alike greater autonomy, and that could be used to alleviate the adverse impact of the coronavirus pandemic in the months to come (Fig. 1).

1. Before any consultation, active COVID-19 infections and suspected cases should be excluded. Testing patients with the use of molecular or serological testing should be weighed on an individual basis.
2. ART treatment should be allowed for eligible (i.e., with no risk factors that might increase COVID-19 morbidity) ‘low prognosis’ patients (e.g., Poseidon groups 2, 3, and 4):
  i. Poseidon group patients 2 could undergo ovarian stimulation and IVF for oocyte freezing or embryo freezing, as appropriate, as the ovarian reserve is still fair [15];
  ii. Poseidon group 3 patients could undergo ovarian stimulation and ART for oocyte freezing or embryo freezing as the rate of oocyte and embryo aneuploidy is relatively low [26];
  iii. For Poseidon group 4 patients, multiple ovarian stimulations (e.g., consecutive stimulation or DuoStim) and fresh or frozen embryo transfer –with or without preimplantation genetic testing (PGT-A)– should be considered, as appropriate [26].
  iv. The ART calculator could be used to estimate the number of oocytes required to achieve at least one euploid embryo for transfer [10, 11].
  v. Personalizing the gonadotropin dose for ovarian stimulation and use of a GnRH antagonist protocol, followed by GnRH agonist triggering, and elective freezing of oocytes or embryos should be the first choice during this emergency period.
  vi. Poseidon groups 2 and 4 patients with comorbidities (e.g., smokers) should be advised to change the lifestyle immediately, as these changes could have a positive impact on overall health and ART success.
3. Fertility preservation should be allowed in women affected by SADs if under clinical remission.
4. Andrological services (e.g., sperm cryopreservation) should be available during any treatment aiming at improving sperm quantity/quality. Thus, we propose that these services are not restricted to oncological patients only.
5. Telehealth and phone counselling for infertile patients should be encouraged.
6. Face to face visits should be limited for infertile patients who demand immediate treatment. Proper personal protective equipment (PPE) should be used by patients and healthcare providers during care provision.
7. ART treatment should be carried out, as much as possible, in free-standing medical facilities, avoiding the diversion of clinical resources, or reduction of hospital capacity that could otherwise be used to care for COVID-19 patients.
8. In closed-controlled air systems, the airflow might produce an increase in the viral spread from potential asymptomatic patients. Thus, special attention should be given to air quality control, including the use of air filtration systems and air pressurization, particularly in surgical and laboratory areas [27].
9. Adherence to regulatory infection prevention recommendations should be of utmost importance for patients and health practitioners [28]. This advice includes the use of appropriate PPE by staff, adherence to social distancing measures for staff and patients, and staggering appointments so that no patients are waiting together in the clinic waiting area.
10. The importance of training of ART staff (clinicians, embryologists, nurses) on PPE needs and usage is stressed.
11. Good standard laboratory practices should be strictly applied when handling gametes and embryos within the embryology and andrology laboratories.
12. A thorough discussion between patients and health care professionals should be made for responsible shared decisions, and psychological support should be an option for those in need.
13. Advanced planning should guide the restart of ART services. Working groups and quality managers should design scenarios on which patients to prioritize, how working lists should be filled and staff scheduling.

### Practical considerations for ART treatment providers

During the coming weeks, we should continue to look critically and dispassionately at the COVID-19 pandemic. Our recommendations are unlikely to create any further burden to the medical infrastructure as ART in the population mentioned above is virtually free of complications. We realize that much is unknown about the implications of COVID-19 for early- and late pregnancy, including maternal-fetal transmission and teratogenicity [29, 30]. However, pregnancy can act as a comorbidity, and we, therefore, currently recommend against conception by ART in most cases. However, it should also be acknowledged that new serology testing is being developed to help identify individuals who have had the infection and have recovered, suggesting that those people are now immune to the virus and could be allowed IVF treatment [31]. Likewise, as IVF health care professionals recover from COVID-19 infections and acquire immunity, there will be less risk of recommencing care, particularly in COVID-19 recovered patients. Along these lines, immune patients will have a low risk of pregnancy complications in the event of an embryo transfer, or of propagating the disease when attending a medical facility. Yet, the accuracy of these tests has been debated and it is still unclear how long any immunity lasts and if reinfection is possible [32].

Given the likelihood that this pandemic will continue for many months ahead, we must keep an open mind, and look for what is - and not for fear of what might be.

### Future perspectives

What we visualize as a future scenario is a gradual restart of “less urgent” infertility treatments, which will follow different phases. The dilemma of how ART services are restarted after the present lockdown is genuine, as every country will follow a different recovery curve. Importantly, it should be considered that contamination of patients and medical staff could occur with COVID-19 when ART care is restarted, particularly in the context of asymptomatic shedding. Thus, we reiterate the recommendations given above that care should only be restarted if social distancing can be maintained, proper PPE be available and used, areas regularly disinfected, and screening for signs and symptoms of the infection undertaken before allowing patients embark on ART treatments.

## Conclusions

This article provides a viewpoint to help both competent authorities and health care providers to better identify priorities and remedies for infertile patients impacted by the COVID-19 pandemic restrictions. In a moment when preventing complications and limiting burdens for national health systems could still represent a relevant issue, the correct prognostic stratification of patients and the identification of more “time-sensitive” cases is crucial for guiding the gradual restart of ART services.

## Supplementary information

**Additional file 1 Supplementary Figure 1**. Logistic regression analysis of 1220 trophectoderm biopsies from 436 patients undergoing intracytoplasmic sperm injection and preimplantation genetic testing for aneuploidy by next-generation sequence testing. The plot depicts the fitted probabilities (with 95% confidence intervals) of blastocyst euploidy as a function of female age. Reprinted with permission of Edizioni Minerva Medica from Esteves et al. (9).

**Additional file 2 Supplementary Figure 2**. The graph shows the percent decrease in the probability of a embryo at the blastocyst stage being euploid, which increases progressively with every year of female age. The percentages shown represent the relative loss from the previous year. Reprinted with permission of Edizioni Minerva Medica from Esteves et al. (9).

**Additional file 3 Supplementary Figure 3.** POSEIDON criteria of low prognosis patients in ART. The novel system relies on female age, ovarian reserve markers, ovarian sensitivity to exogenous gonadotropin, and the number of oocytes retrieved, which will both identify the patients with low prognosis and stratify such patients into one of four groups of women with “expected” or “unexpected” impaired ovarian response to exogenous gonadotropin stimulation. According to these criteria, four distinct groups of low prognosis patients can be established (left). Group 1: Patients < 35 years with sufficient prestimulation ovarian reserve parameters (AFC ≥5, AMH ≥1.2 ng/mL) and with an unexpected poor or suboptimal ovarian response. This group is further divided into subgroup 1a, constituted by patients with fewer than four oocytes, and subgroup 1b, constituted by patients with four to nine oocytes retrieved after standard ovarian stimulation. Group 2: Patients ≥35 years with sufficient prestimulation. Ovarian reserve parameters (AFC ≥5, AMH ≥1.2 ng/mL) and with an unexpected poor or suboptimal ovarian response. This group is further divided into subgroup 2a, constituted by patients with fewer than four oocytes, and subgroup 2b, constituted by patients with four to nine oocytes retrieved after standard ovarian stimulation. Group 3: Patients < 35 years with poor ovarian reserve prestimulation parameters (AFC < 5, AMH < 1.2 ng/mL). Group 4: Patients ≥35 years with poor ovarian reserve prestimulation parameters (AFC < 5, AMH. < 1.2 ng/mL). Owing to low oocyte numbers and less embryos produced, POSEIDON patients have lower cumulative live birth rates per started cycle than non-POSEIDON counterparts. However, the prognosis is differentially affected by oocyte quantity and female age, as the latter relates to the risk of embryo aneuploidy (right). Art drawing by Chloé Xilinas. Reprint from Esteves et al. (4). This is an open-access article distributed under the terms of the Creative Commons Attribution License (CC BY).

## Data Availability

Not applicable.

## Abbreviations

ART: Assisted reproductive technology
CI: Confidence interval
COVID-19: Coronavirus disease 2019
IUI: Intrauterine insemination
IVF: In vitro fertilization
POSEIDON: Patient-oriented strategies encompassing individualized oocyte number
SADs: Systemic autoimmune diseases
