# Supplementary figures and images for: COVID-19 and assisted reproductive technology services: repercussions for patients and proposal for individualized clinical management
Source: Reprod Biol Endocrinol. 2020 May 13;18:45. doi: 10.1186/s12958-020-00605-z (PMC7218705; doi:10.1186/s12958-020-00605-z)

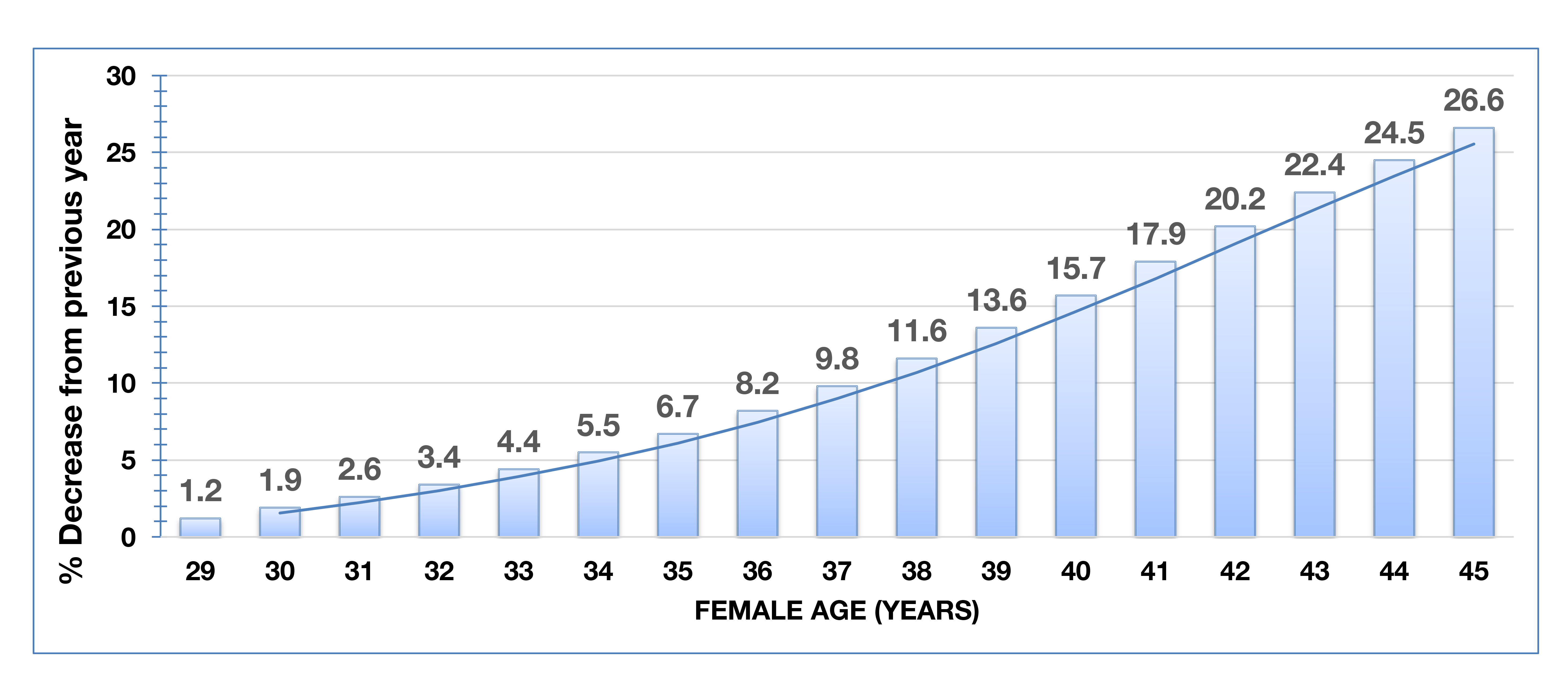

Supplement: Supplementary file 2 — Additional file 2 Supplementary Figure 2. The graph shows the percent decrease in the probability of a embryo at the blastocyst stage being euploid, which increases progressively with every year of female age. The percentages shown represent the relative loss from the previous year. Reprinted with permission of Edizioni Minerva Medica from Esteves et al. (9). [file 12958_2020_605_MOESM2_ESM.jpg]
